# Multimodal Diffusion-MRI and MEG Assessment of Auditory and Language System Development in Autism Spectrum Disorder

**DOI:** 10.3389/fnana.2016.00030

**Published:** 2016-03-23

**Authors:** Jeffrey I. Berman, James C. Edgar, Lisa Blaskey, Emily S. Kuschner, Susan E. Levy, Matthew Ku, John Dell, Timothy P. L. Roberts

**Affiliations:** ^1^Department of Radiology, Children's Hospital of PhiladelphiaPhiladelphia, PA, USA; ^2^Department of Radiology, Perelman School of Medicine, University of PennsylvaniaPhiladelphia, PA, USA; ^3^Department of Pediatrics, Children's Hospital of PhiladelphiaPhiladelphia, PA, USA

**Keywords:** autism spectrum disorders (ASD), multimodal imaging, magnetoencephalography (MEG), diffusion MRI, auditory pathways, language

## Abstract

**Background:** Auditory processing and language impairments are prominent in children with autism spectrum disorder (ASD). The present study integrated diffusion MR measures of white-matter microstructure and magnetoencephalography (MEG) measures of cortical dynamics to investigate associations between brain structure and function within auditory and language systems in ASD. Based on previous findings, abnormal structure-function relationships in auditory and language systems in ASD were hypothesized.

**Methods:** Evaluable neuroimaging data was obtained from 44 typically developing (TD) children (mean age 10.4 ± 2.4 years) and 95 children with ASD (mean age 10.2 ± 2.6 years). Diffusion MR tractography was used to delineate and quantitatively assess the auditory radiation and arcuate fasciculus segments of the auditory and language systems. MEG was used to measure (1) superior temporal gyrus auditory evoked M100 latency in response to pure-tone stimuli as an indicator of auditory system conduction velocity, and (2) auditory vowel-contrast mismatch field (MMF) latency as a passive probe of early linguistic processes.

**Results:** Atypical development of white matter and cortical function, along with atypical lateralization, were present in ASD. In both auditory and language systems, white matter integrity and cortical electrophysiology were found to be coupled in typically developing children, with white matter microstructural features contributing significantly to electrophysiological response latencies. However, in ASD, we observed uncoupled structure-function relationships in both auditory and language systems. Regression analyses in ASD indicated that factors other than white-matter microstructure additionally contribute to the latency of neural evoked responses and ultimately behavior. Results also indicated that whereas delayed M100 is a marker for ASD severity, MMF delay is more associated with language impairment.

**Conclusion:** Present findings suggest atypical development of primary auditory as well as auditory language systems in ASD. Findings demonstrate the need for additional multimodal studies to better characterize the different structural features (white matter, gray matter, neurochemical concentration) that contribute to brain activity, both in typical development and in ASD. Finally, the neural latency measures were found to be of clinical significance, with M100 associated with overall ASD severity, and with MMF latency associated with language performance.

## Introduction

The etiology or, indeed, etiologies of autism spectrum disorder (ASD) is currently unknown. It is hypothesized that alterations to brain structure and function contribute to the clinical symptoms common to ASD. Given the high rate of occurrence of communication and language impairments in ASD, research has focused on the brain regions associated with basic auditory processes and more complex language skills, with prior imaging studies showing alterations in temporal lobe structure, connectivity and function (Klin et al., 2002; Boddaert et al., 2004; Redcay and Courchesne, 2005; Lee et al., 2007; Lange et al., 2010; Schipul et al., 2011; Nickl-Jockschat et al., 2012; Roberts et al., 2013). Although, atypical development of temporal regions is believed to precede and possibly underlie language impairments in ASD (Wolff et al., 2012; Edgar et al., 2015a), the links between abnormal development of brain structure and function with the behavioral phenotype of ASD are poorly understood.

To investigate associations between structure and function within auditory and language systems in ASD, the present study integrated diffusion magnetic resonance imaging (dMRI) measures of white-matter microstructure with magnetoencephalography (MEG) measures of cortical neural dynamics. Diffusion MRI is sensitive to microstructural properties of white matter such as axon density and myelination and has been used in quantitative studies of the superior temporal gyrus and arcuate fasciculus in ASD (Lee et al., 2009; Nagae et al., 2012; Berman et al., 2013). MEG is a non-invasive neuroimaging modality that records neuronal currents with high temporal resolution and has been used in many studies to examine the neural dynamics of auditory encoding processes in ASD (Wilson et al., 2007; Roberts et al., 2010; Stroganova et al., 2013). In the present study, associations between the rate of encoding auditory information and the structural integrity of two white-matter tracks were examined. The white matter and cortical neural measures selected for this study followed the propagation of auditory input from basic auditory encoding processes (primary/secondary auditory cortex and auditory radiations) to higher-level auditory linguistic processes (vowel mismatch discrimination and arcuate fasciculus). To examine maturation of these measures, cross-sectional associations with age were also examined.

The first white-matter track examined was the auditory radiation, the pathway that relays acoustic information from the medial geniculate nucleus (MGN) of the thalamus to the primary/secondary auditory cortex of the superior temporal gyrus (STG). The second white matter pathway examined was the arcuate fasciculus (AF), a tract from the STG to higher-order language areas. These circuits represent relatively early stages of connection relevant for auditory processing and then later stages relevant for language functioning. To this end, the first functional MEG measure examined was the 100 ms (M100) STG auditory response. Auditory evoked M100 latency has been shown to be due, in part, to thalamocortical conduction velocity along the auditory radiation (Roberts et al., 2009, 2013). The second functional measure examined was the auditory mismatch field (MMF) elicited in response to an odd-ball stimulus among a series of otherwise similar stimuli (e.g., the “/u/” in “/a//a//a//a//u//a/”). The MMF response is involved in passive sound processing and is a precursor to language processing (Näätänen et al., 2007). Thus, M100 latency is considered to reflect relatively basic auditory encoding processes and MMF latency an index of preparatory language function.

This study was motivated by prior studies that individually focused on auditory or language systems in ASD (Oram Cardy et al., 2005; Roberts et al., 2010, 2013). For example, the latency of the auditory M100 decreases between infancy and adulthood, with M100 latency delays, indicating slower conduction and processing of auditory stimuli, observed in ASD (Rojas et al., 1998; Wunderlich et al., 2006; Roberts et al., 2010). The latency of the MMF has also been examined and appears to be a marker of language impairment, with MMF latency delays observed in individuals with specific language impairment (SLI) and associated with language impairment in ASD (Roberts et al., 2011).

Recent studies combining MEG and diffusion MR have shown associations between M100 conduction velocity and Heschl's gyrus white-matter integrity in typically developing (TD) children, with these associations less evident in children with ASD (Roberts et al., 2013). The present study extended prior studies from our laboratory to support the hypothesis that abnormal structure–function relationships are pervasive across auditory and language systems. To this end, high angular resolution diffusion imaging (HARDI) tractography was employed to measure the microstructural integrity of the entire auditory radiation to assess associations between auditory radiation tract microstructure and M100 latency. Similarly, diffusion MR white-matter measures of the arcuate fasciculus were obtained and associations with vowel-contrast MMF latency examined.

## Materials and methods

### Participants

Children with a prior clinical ASD diagnosis were recruited from the Regional Autism Center of The Children's Hospital of Philadelphia (CHOP) and from local parent support groups. During the research visit, clinical and diagnostic testing by licensed child psychologists were performed to confirm ASD diagnosis and to ensure that typically developing children met inclusion criteria. ASD diagnosis was confirmed with the *Autism Diagnostic Observation Schedule (ADOS;* Lord et al., 2000) and parent report on the *Social Communication Questionnaire (SCQ;* Rutter et al., 2003) and the *Social Responsiveness Scale (SRS;* Constantino et al., 2003). In the rare event that diagnosis could not be confirmed with the ADOS and parent questionnaires alone, the Autism Diagnostic Interview-Revised (ADI-R) was administered to resolve diagnostic discordances (Lord et al., 1994). To diagnose language impairment (LI), all subjects were evaluated with the *Clinical Evaluation of Language Fundamentals – 4th edition* (*CELF-4;* Semel et al., 2003). Language impairment (LI) was defined based on the CELF-4 core language index, using a threshold of at or below 1SD from the mean and the 16^th^ percentile (i.e., SS < 85).

Inclusion criteria for the typically developing children included having no history of neurodevelopmental, psychiatric, or neurological disorders, scoring below cut-offs for ASD on the ADOS and on parent questionnaires, and performing above the 16^th^ percentile on the CELF-4. All subjects scored at or above the second percentile (SS > 70) on either the Perceptual Reasoning Index (PRI) or the Verbal Comprehension Index (VCI) of the Wechsler Intelligence Scale for Children-IV (WISC-IV). Detailed inclusion and exclusion criteria of the ASD and TD groups have been described previously (Roberts et al., 2010). The study was approved by the CHOP Institutional Review Board and all participants' legal guardian(s) gave informed written consent. Where competent to do so, children over 7 years gave verbal assent.

The pool of participants with evaluable neuroimaging data for this multimodal study included 44 TD (mean age 10.4 ± 2.4 years) and 95 ASD (mean age 10.2 ± 2.6 years). MEG and DTI findings from a smaller subset of these participants have been previously reported (Roberts et al., 2010, 2011). Group differences in age were not significant [*t*-test, *t*_(127)_ = 0.35, *p* = 0.70]. Of the children with ASD with diagnostic language scores, 35 were classified as ASD with language impairment (ASD/+LI; mean age 9.3 ± 2.5 years) and 56 as ASD without language impairment (ASD/-LI; mean age 10.6 ± 2.5 years). Although, the difference in age between ASD/+LI and ASD/-LI was significant (*t*-test, *p* = 0.04), the age difference was small. Given the challenges of multimodal imaging, complete diffusion MR and MEG datasets were not available for all subjects. In particular, 28 participants had evaluable HARDI and DTI for measurement of the auditory radiation as well as evaluable M100 latency. Eighty-two participants had evaluable MMF latency and 78 participants had evaluable DTI for measurement of the arcuate fasciculus.

### MEG

Data were acquired in a magnetically shielded room using a 275-channel whole-cortex CTF magnetometer (VSM MedTech Inc., Coquitlam, BC). The details of the M100 and MMF tasks and data processing have been previously described (Roberts et al., 2010, 2011). In brief, for the M100 task, tones of 200, 300, 500, and 1000 Hz (300 ms duration, 10 ms ramps) were passively presented at 45 dB sensation level and repeated 130 times. The inter-stimulus interval varied randomly between 900 and 1100 ms. The left and right STG M100 latency response was obtained using a left and right STG dipole source model that transformed MEG sensor signals into source space. For the MMF task, auditory stimuli consisted of the vowels /a/ and /u/ (300 ms in duration), with deviant stimuli occurring at random positions in the sequence with a 15% probability. Inter-stimulus interval was 700 ms. Two runs with the vowels alternating as standard/deviant allowed matched token subtraction (i.e., deviant /u/–standard /u/). MMF latency, again obtained using a left and right STG dipole source model, was defined at the maximal deflection in the difference waveform, occurring between 150 and 350 ms after stimulus onset, and after identifiable M50 and M100 components in the unsubtracted waveforms.

### MR imaging

MR imaging was performed with a 3T Siemens Verio™ and 32-channel head coil (Siemens Medical Solutions, Erlangen, Germany). HARDI and conventional DTI were performed. Whole-brain HARDI acquisition included 64 gradient directions at *b* = 3000 s/mm^2^, *TR*/*TE* = 14.8 s/111 ms, voxel size = 2 × 2 × 2 mm, and 128 × 128 matrix. DTI acquisition used 30 diffusion gradient directions at *b* = 1000 s/mm^2^, one *b* = 0 s/mm^2^ volume, *TR*/*TE* = 11 s/76 ms, voxel size 2 × 2 × 2 mm, and 128 × 128 matrix. The HARDI acquisition was 18 min in duration and the DTI acquisition 6 min in duration. Anatomical T1-weighted MP-RAGE volumes were also acquired with TR/TE/TI = 1900/2.87/1050 ms, 1 mm isotropic voxels, and full head coverage.

**Figure 1:**
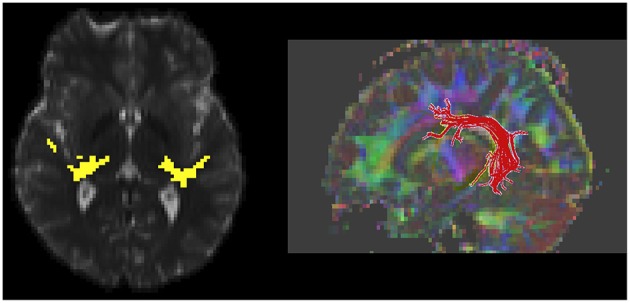
**White matter measurement regions of interest, as defined with diffusion MR tractography, are shown**. An axial slice through the auditory radiation tracts shows connectivity from the auditory cortex to thalamus (yellow, **left**). A 3D rendering of left-hemisphere arcuate fasciculus is shown (**right**).

Solid angle q-ball reconstruction of the HARDI data was used with a probabilistic fiber tracking algorithm to delineate the left and right auditory radiation (Figure 1; Berman et al., 2008, 2013). Although, difficult to obtain in children, HARDI tractography is necessary to traverse the crossing fibers of the auditory radiation (Berman et al., 2013). In contrast, DTI deterministic fiber tracking is sufficient to follow the core of the left and right arcuate fasciculus tracts (Figure 1; Mori et al., 1999). The arcuate fasciculus was selected based on previous reports and by *a priori* hypothesis to reduce statistical confounds associated with multiple comparisons in more comprehensive explorations (Nagae et al., 2012). DTI parameters (fractional anisotropy “FA,” mean diffusivity “MD,” radial diffusivity “RD,” and axial diffusivity “AD”) were determined from the eigen-values of the diffusion tensor and evaluated voxelwise over the course of the left and right auditory radiations and arcuate fasciculus as determined with tractography.

### Statistical analysis

Statistical analyses included group comparisons, multivariate regression, and linear mixed models. Analyses were performed using JMP (Version 11, SAS). A single “effective” M100 latency for each participant's left and right STG was calculated with a linear mixed model to reduce the number of M100 latency analyses (Roberts et al., 2000). Given that M100 latency varies by stimulus tone frequency, the “effective” M100 latencies were computed with a linear mixed-model. The M100 latency model contained fixed effects of hemisphere, stimulus frequency, group, age, and a random effect of subject. Fitted values from the model were used to predict M100 latency for participants with partially missing observations (e.g., absent response to a stimulus tone and/or in one hemisphere) to enable comparisons across participants with different distributions of missing data. Considering subject as a random effect (on the model intercept) allows retention of inter-individual differences when modeling and imputing missing data.

## Results

### Auditory system: Auditory radiation and M100

Each DTI parameter was linearly modeled with main effects of group, age, and hemisphere, along with each two-way interaction term. Auditory radiation MD and RD decreased with age and FA increased with age, indicating white matter maturation across both ASD and TD in the studied age range [MD: *F*_(1, 52)_ = 9.1, *p* = 0.004; RD: *F*_(1, 52)_ = 11.8, *p* = 0.001; FA: *F*_(1, 52)_ = 6.63, *p* = 0.013]. The main effect of group was not significant for any DTI parameter. Significant age by group interactions for FA and RD [FA: *F*_(1, 52)_ = 4.66, *p* = 0.036; RD: *R*_(1, 52)_ = 4.85, *p* = 0.032], indicated group differences in maturation. In particular, collapsing across hemisphere, FA increased by 0.013 per year (95^th^ CI [0.003, 0.02]) in TD vs. 0.001 per year (95^th^ CI[–0.005, 0.007]) in ASD, and RD decreased by 15.2 × 10^−6^ mm^2^/s/year (95^th^ CI [−25, −46] × 10^−6^) in TD vs. 3.04 × 10^−6^ mm^2^/s/year (95^th^ CI [−9, 2.5] 10^−6^) in ASD.

**Figure 2:**
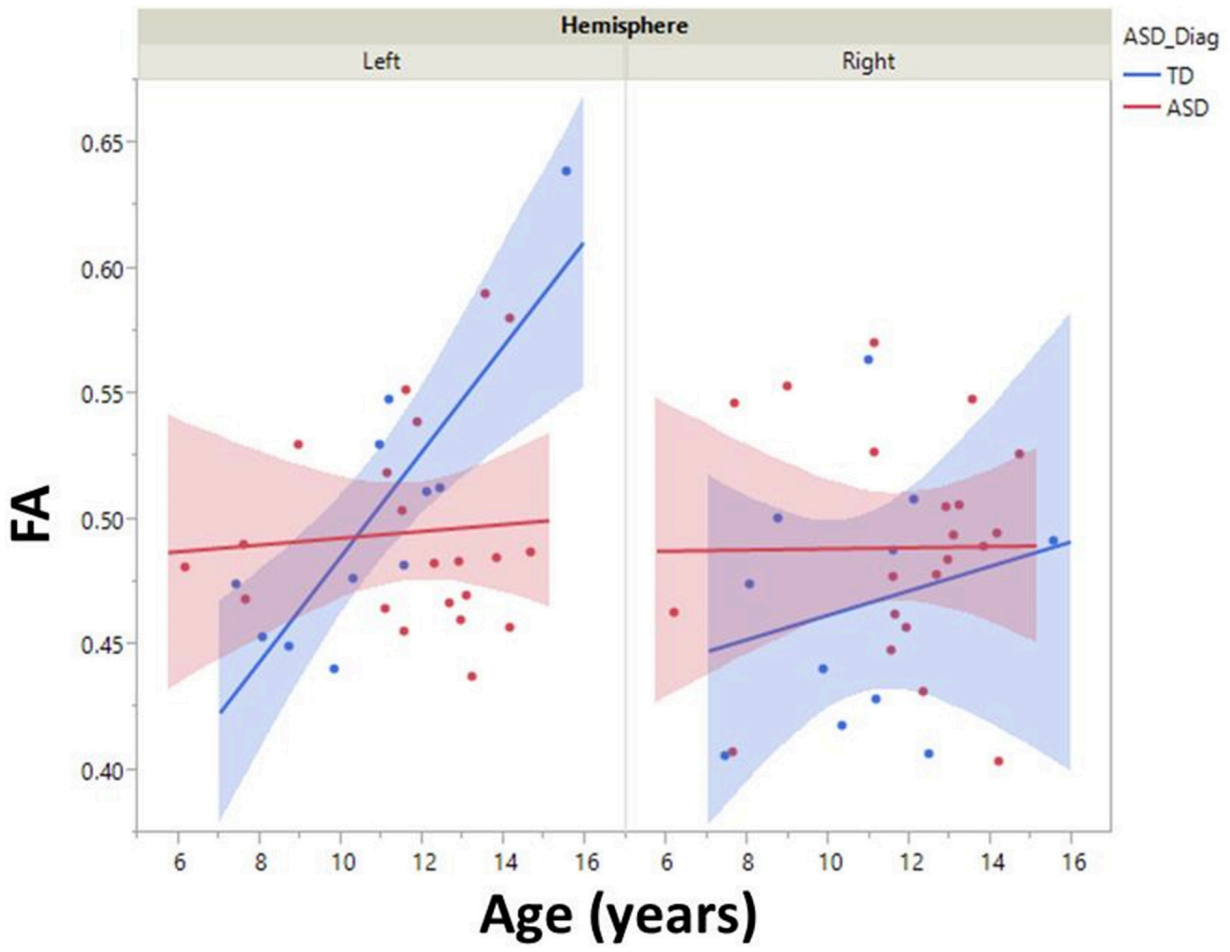
**Developmental trajectory of left and right auditory radiation microstructure are shown with 95% confidence intervals (shading)**. Left-hemisphere FA increased at a faster rate in the TD vs. ASD group (*p* < 0.01). No right-hemisphere group difference in maturation was observed.

Although, there was no significant group by hemisphere by age interaction, given overall group maturation rate differences and *a priori* hypotheses relating to the development of lateralized hemisphere functional specialization, left and right hemisphere DTI parameters were assessed separately. In the left hemisphere, significant group by age interaction terms indicated group differences in the maturation of left hemisphere FA [*F*_(1, 26)_ = 7.9, *p* = 0.01], RD [*F*_(1, 26)_ = 6.2, *p* = 0.02], and AD [*F*_(1, 26)_ = 5.4, *p* = 0.03]. As depicted in Figure 2, *left hemisphere* FA increased at a faster rate in the TD than ASD, driving the overall group by age difference. The slower maturation of FA in ASD was likely due to a slower rate of RD decrease in ASD vs. TD. No differences in rate of maturation were detected in the right hemisphere.

**Figure 3:**
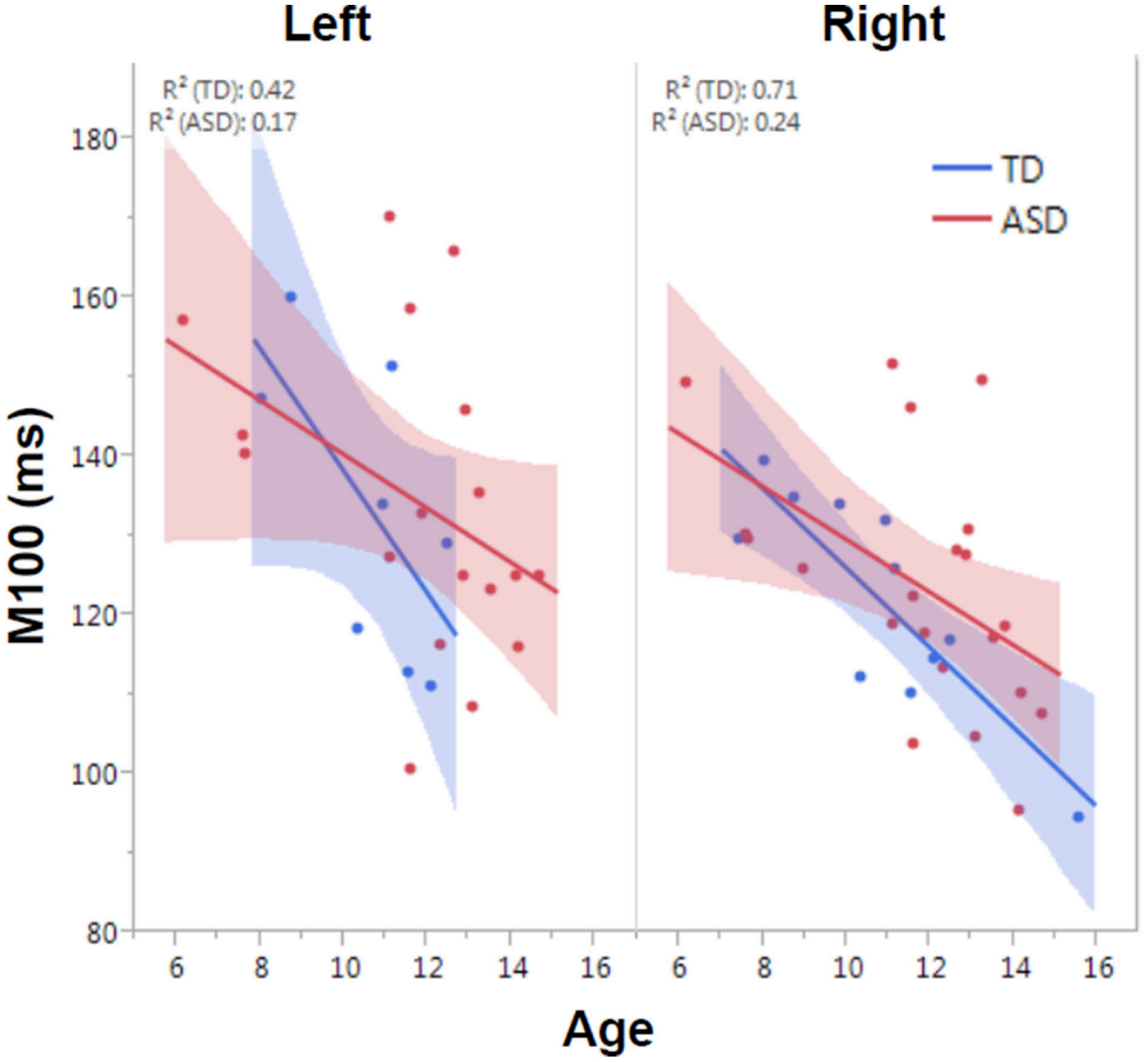
**Developmental trajectory of left and right auditory M100 latency**. M100 latency shortened with age across the population (*p* < 0.0001). Although no group difference in rate of maturation was observed, the TD group trended toward faster maturation.

**Figure 4:**
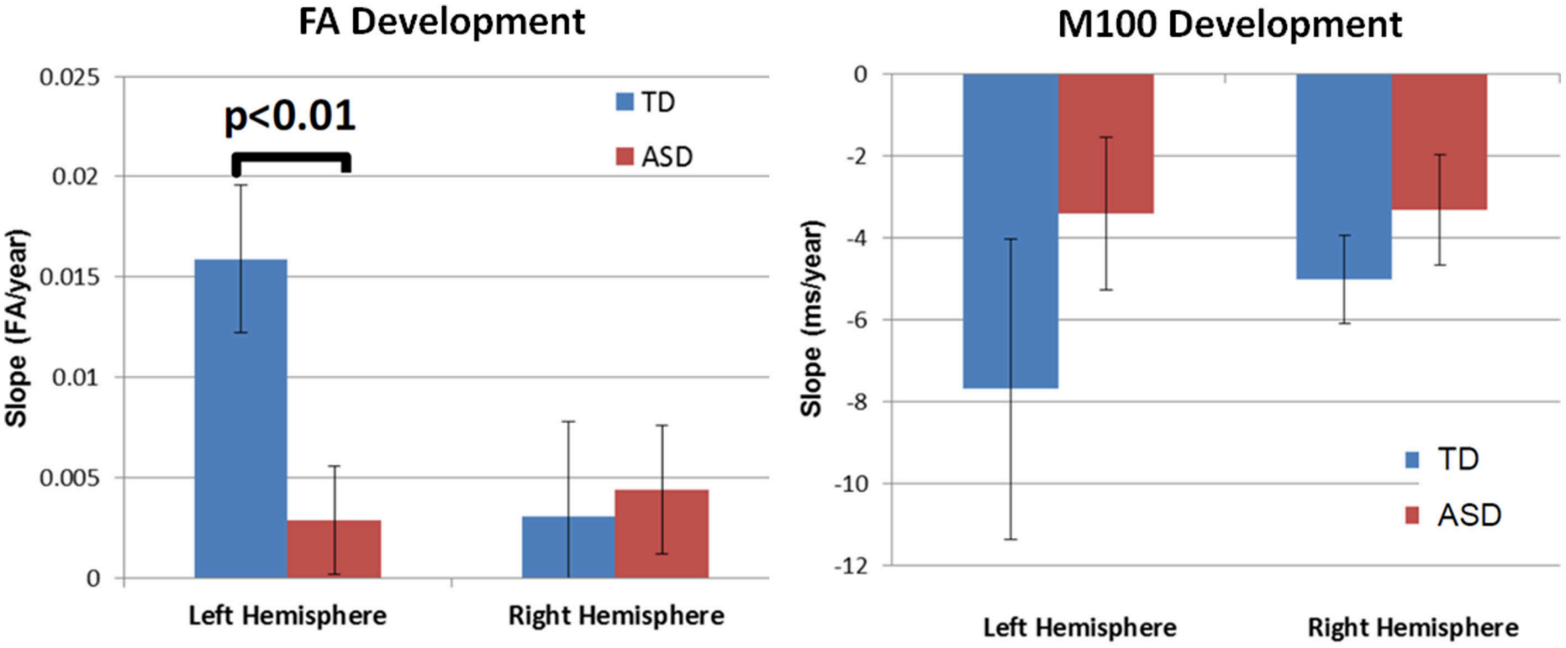
**Developmental trajectory (slope) of auditory radiation FA (left) and auditory cortex M100 latency (right)**. Left-hemisphere FA development was significantly slower in ASD vs. TD (*p* < 0.01). Although not reaching significance, the left-hemisphere M100 latency showed a similar pattern of more rapid development in TD vs. ASD. For both MEG and DTI measures, a lack of hemispheric asymmetry or specialization is evident in the ASD group.

M100 was similarly modeled with group, age and hemisphere as main effects, as well as each interaction term. A main effect of hemisphere, *F*_(1, 52)_ = 8.0, *p* = 0.01, indicated that M100 responses were 11 ms later in the left than right hemisphere, consistent with prior reports (Paetau et al., 1995; Howard and Poeppel, 2009). A main effect of age, *F*_(1, 52)_ = 28.6, *p* < 0.0001, showed that M100 decreased ~4.5 ms per year (Figure 3). No group difference in rate of maturation was detected. Again, despite the absence of a group by hemisphere interaction, *a priori* hypotheses called for the interrogation of M100 latency maturation in each hemisphere. Figure 4 compares the left and right hemisphere maturation rates for FA and M100. Left-hemisphere M100 latency tended to decrease at a faster rate in TD than ASD. Although, the group difference in left hemisphere M100 maturation did not reach significance, this group difference pattern is conspicuously similar to the significant group differences in maturation of left hemisphere FA. Also of note, although in this sample groups did not differ in M100 latency, Figure 3 suggests later right-hemisphere M100 responses in ASD vs. TD, a pattern consistent with prior reports (Roberts et al., 2010; Edgar et al., 2015b).

**Figure 5:**
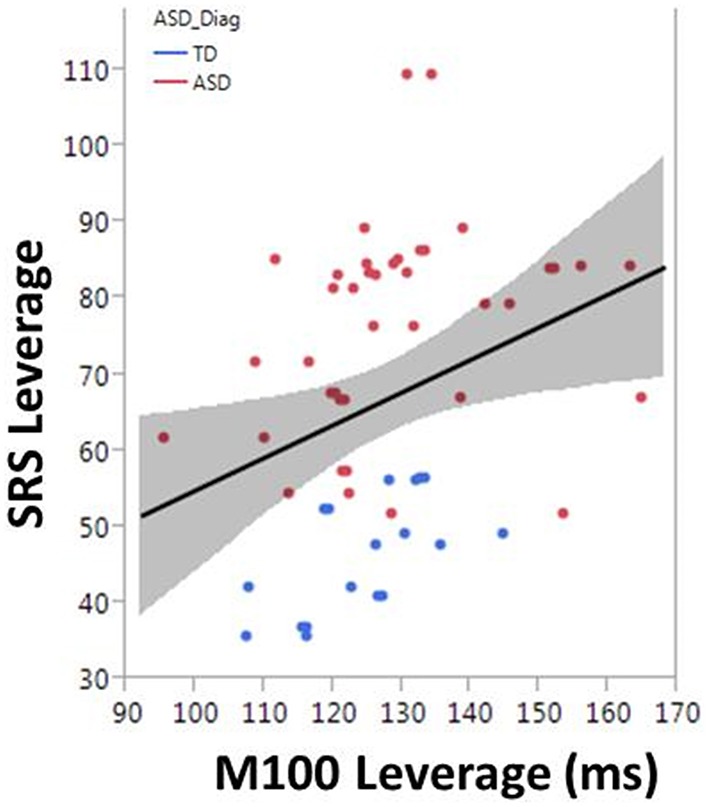
**The correlation between M100 and SRS is observed in the partial regression leverage plot**. The ordinate (y-axis) of the leverage plot shows the residuals of the response variable (SRS) when regressed on all model parameters except M100. The abscissa (x-axis) of the leverage plot shows the residuals from regressing M100 against the other independent variables. M100 is a significant predictor of SRS (*p* = 0.02).

To examine the association between M100 and behavior, ASD symptom and language ability measures (SRS and CELF-4) were modeled with M100 latency, age, and hemisphere as factors. M100 was a significant predictor of SRS [*F*_(1, 57)_ = 6.1, *p* = 0.02]. M100 did not predict language ability (CELF-4). A mean adjusted leverage plot was used to isolate and visualize the relationship between SRS and M100 latency (Figure 5).

For the multimodal analysis, to account for additional variance in the M100 latency, a linear model of each participant's effective M100 latency with average DTI parameters and group as effects was constructed. Age was not included given the above results indicating that both DTI and M100 measures show age dependence and may have interdependent maturational trajectories. A main effect of RD, *F*_(1, 27)_ = 4.7, *p* = 0.04, showed that increased RD was related to longer M100 latencies (slope = 15.7 × 10^4^ ms ^*^ s/mm^2^). A similar trend was observed between MD and M100 latency, *F*_(1, 27)_ = 3.4, *p* = 0.08. Analyzing groups separately showed this structure-function relationship was primarily driven by the TD group: RD was positively correlated with M100 in TD, *F*_(1, 8)_ = 6.2, *p* = 0.04 (slope = 20.2 × 10^4^ ms ^*^ s/mm^2^, 95^th^ CI: [1.4, 39] × 10^4^), but not in ASD, *F*_(1, 18)_ = 0.8, *p* = 0.4 (slope = 10.4 × 10^4^ ms ^*^ s/mm^2^).

### Language system: MMF latency and arcuate fasciculus

Given the reported relationship between MMF latency and language ability, group comparisons of CELF-4 and MMF latency included three groups: ASD with and without language impairment (ASD/+LI, ASD/-LI) as well as TD. As expected, mean CELF-4 Core Language Index (CLI) scores were significantly different for all pairwise group comparisons (TD: 109 ± 11.9; ASD/-LI: 98.6 ± 10.2; ASD/+LI: 70.7 ± 14.6; *p*'s < 0.005 with Tukey–Kramer Adjustment for multiple comparisons). MMF latency was modeled with group, stimulus type (vowel /a/ or /u/), and hemisphere as fixed effects and subject as a random effect. Additionally, PRI was considered as a potential confounding covariate.

**Figure 6:**
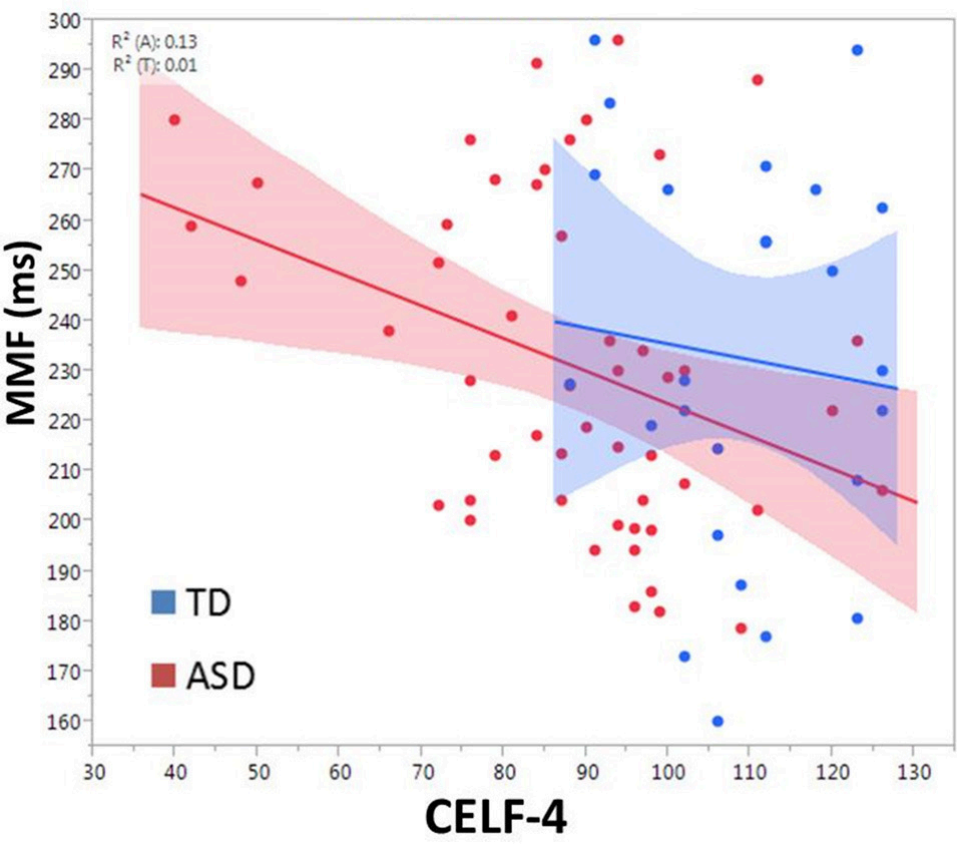
**Relationship between CELF-4 score and MMF latency is shown for ASD (red) and TD (blue)**. The correlation is significant for ASD (*p* < 0.005). Although not significant in TD, the slope suggests a negative correlation between MMF and language ability.

There was a main effect of group on MMF latency, *F*_(2, 72.4)_ = 4.2, *p* = 0.02 (mean TD: 229.9 ± 6.7 ms; ASD/-LI: 220.1 ± 5.9 ms; ASD/+LI: 248.3 ± 8.1 ms; Roberts et al., 2011). ASD/+LI exhibited MMF latencies ~25 ms later than ASD/-LI (*p* < 0.02, Tukey–Kramer Adjustment for multiple comparisons). MMF latency did not differ with respect to stimulus type or hemisphere and was not associated with PRI or age. Within the combined ASD group, MMF latency (latency collapsed across hemisphere) correlated with CELF-4 CLI, *F*_(1, 49.4)_ = 8.4, *p* = 0.01 (Figure 6). Separately examining the left and right hemisphere, in ASD, both left and right hemisphere MMF latency correlated with language ability, *F*_(1, 43.5)_ = 7.5, *p* = 0.01 in left; *F*_(1, 42.8)_ = 6.1, *p* = 0.01 in right. No significant association between MMF latency and language ability (CELF-4 CLI) was observed in TD. In contrast to M100 latency, no significant association between MMF latency and SRS was observed in ASD or TD.

To examine the role of white-matter microstructure on MMF latency as well as language ability, arcuate fasciculus DTI parameters were measured. Arcuate fasciculus FA increased with age in the ASD, *F*_(1, 85)_ = 4.0, *p* = 0.05 (slope = 0.0024/year) and TD groups, *F*_(1, 55)_ = 21.8, *p* < 0.0001 (slope = 0.0052/year). MD decreased with age in the ASD, *F*_(1, 85)_ = 22.4, *p* < 0.0001 (slope = −0.052 × 10^−4^ mm^2^/s/year) and TD groups *F*_(1, 55)_ = 28.6, *p* < 0.0001 (slope = −0.076 × 10^−4^ mm^2^/s/year). Group by age interactions were not significant, indicating no detectable group difference in FA or MD maturation slopes. DTI parameters (with age as a covariate) were not predictive of CELF-4 CLI in either ASD or TD.

**Figure 7:**
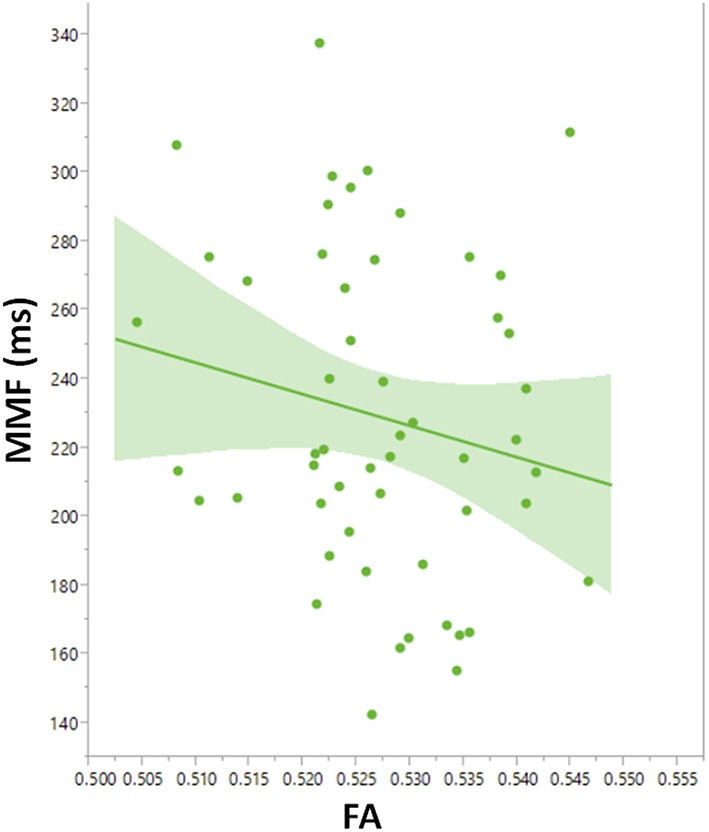
**Leverage plot of arcuate fasciculus FA vs. MMF latency in TD showing a significant correlation (***p*** < 0.01)**. Left and right hemisphere measures are combined in the scatter plot.

To explore contributions to MMF latency by arcuate fasciculus microstructure, linear mixed models with MMF latency as the dependent variable were constructed with DTI parameter, PRI, group (ASD and TD), age, stimulus type, and hemisphere as main effects and subject as the random effect. A main effect of FA, *F*_(1, 103)_ = 4.2, *p* = 0.04, indicated associations between FA and MMF latency. Explored in each group, FA predicted MMF in TD, *F*_(1, 45.8)_ = 4.60, *p* = 0.04 (Figure 7), and not in ASD (*p* = 0.3) or in the ASD/+LI (*p* = 0.4) or ASD/-LI (*p* = 0.4) subgroups. No associations with MMF latency were observed for the other DTI parameters (MD, RD, and AD; *p* > 0.05).

## Discussion

The present multimodal and multi-circuit study examined associations between brain structure and function in auditory and language areas. In both auditory and language systems, white-matter integrity, and cortical electrophysiology were found to be coupled in TD, with white-matter microstructural features contributing to M100 and MMF latency. However, in ASD, these structure–function relationships were less obvious or uncoupled. Results also suggested that the neural latency measures are of clinical significance, with a delayed M100 associated with increased ASD severity (as measured with SRS) and a delayed MMF delay associated with greater language impairment (CELF-4 CLI).

Accurate and rapid encoding of auditory information is critical for receptive language. A prior study of auditory processing observed abnormal brainstem and cortical electrophysiology in children with language learning problems (Wible et al., 2005). M100 latency provides information about the average auditory pathway conduction velocity to primary/secondary auditory areas, with delayed auditory encoding reported in ASD (Roberts et al., 2010). In the present study, M100 latency was observed to shorten with age in both groups. Regarding brain structure, diffusion MR analyses also demonstrated maturation of the thalamocortical segment of the central auditory pathway in TD and ASD. As the midbrain white matter and vestibulocochlear nerve portions of the auditory system were not examined, it remains undetermined if the thalamocortical measurements are representative of the entire auditory pathway. Despite observing maturation of white-matter measures and M100 latency in ASD, analyses indicated slower maturation in ASD vs. TD, especially in the left hemisphere. Although, maturation of conduction efficiency occurs in ASD, there was some suggestion of a lateralized abnormality in ASD, with left-hemisphere maturation of FA slower in ASD vs. TD. Additionally, in the TD group, a hemisphere asymmetry in microstructural development trajectory was observed, consistent with the known structural and volumetric asymmetries of the auditory and language systems (Geschwind and Levitsky, 1968; Morillon et al., 2010). The children with ASD, however, did not exhibit this hemispheric asymmetry, consistent with prior reports of abnormal hemispheric asymmetry of STG and arcuate fasciculus white matter in ASD (Fletcher et al., 2010; Lange et al., 2010). Mirroring structural maturation, M100 latency results also indicated a lack of hemispheric asymmetry in ASD. In particular, although not reaching significance, the rate of left hemisphere M100 latency shortening appeared diminished in children with ASD vs. TD. Given that in ASD the FA and M100 analyses both suggested a loss of the hemispheric asymmetry observed in TD, present findings lend support to the hypothesis of a lack of hemispheric functional specialization in ASD. It should be noted that many studies with methods ranging from structural to functional support the atypical development of left and right hemispheres in ASD (Lange et al., 2010; Herbert et al., 2002, 2005; De Fossé et al., 2004; Flagg et al., 2005).

Coupling of structure and function in typically developing children was evidenced by associations between auditory radiation and arcuate fasciculus white-matter microstructure and M100 and MMF latency. In ASD, however, white-matter microstructure was not predictive of either M100 or MMF latency. A prior study observed a similar structure-function uncoupling in ASD when examining the latency of the earlier M50 auditory response and Heschl's Gyrus white-matter microstructure (Roberts et al., 2013). Of note, in the present study, the specificity of “auditory radiation to M100” and “arcuate fasciculus to MMF” coupling was established by considering cross-regional correlations of M100 to arcuate microstructure and MMF to auditory radiation microstructure, with each of these pairings found to be unrelated (although with findings limited by the number of subjects with complete data sets).

Appropriate white matter structural maturation appears necessary, but not sufficient for efficient auditory conduction and processing. Other factors that contribute to M100 and MMF latency likely include cortical architecture and synaptic transmission. Age-related changes in synaptic efficiency and cortical layers are associated with maturation of the auditory response (Steinschneider et al., 1994; Eggermont and Ponton, 2003). Pyramidal cells are the primary source of MEG activity and maturation of these neurons can modulate MEG recording (Lewine and Orrison, 1995; Spruston, 2008; Elston et al., 2010; Lange et al., 2010; Elston and Fujita, 2014). As an example, studies have noted associations between gray matter cortical thickness and the strength of auditory responses (Edgar et al., 2012). Future studies examining the contributions of white matter, gray matter, and neurochemistry to M100 and MMF latencies in ASD are needed.

In the present study, M100 latency was associated with the severity of autism symptoms, as assessed by the SRS. M100 latency was not associated with language performance. In contrast, the observed difference in MMF latency between ASD subjects with and without language impairment, as well as the bilateral associations of MMF latency and CELF-4 performance (and the concomitant lack of MMF association with SRS or PRI), supported the hypothesis that MMF latency is an index of language impairment rather than ASD severity or intellectual ability (Roberts et al., 2011). An association of MMF latency with language ability only in ASD suggested that MMF latency tracks with language *impairment* and not the normal variation in CELF-4 CLI scores observed in the controls. MMF does not predict language *abilities* in excess of the CELF-4 median. Impaired auditory perception and auditory discrimination are expected to have upstream ramifications for language cognition (Bishop, 2007). The MMF latency and language ability associations in both hemispheres suggests that passive auditory change detection is neither a very basic sensory function nor a higher-level language process restricted to the presumed language-dominant left hemisphere. Basic auditory detection, as measured with the M100, has not been correlated with language ability in this or prior studies. DTI parameters were not directly related to CELF-4 scores in this study. However, as previously reported, when our dataset is not limited to those subjects with both evaluable DTI and MEG data, arcuate fasciculus diffusivity was observed to correlate with CELF-4 scores (Roberts et al., 2014).

A limitation of this and other multimodal studies included incomplete or even sparse datasets. As the number of independent measures or modalities increases, the number of participants expected to have complete data sets unfortunately decreases. It is also of note that although Figure 3 suggested delayed right STG M100 latencies in ASD vs. TD, this group difference was not statistically significant. Failure to replicate previous studies showing right STG M100 latency group differences is likely due to a lack of power given much smaller samples in the present study vs. previous studies.

To conclude, multimodal examination of auditory and language systems in ASD indicated atypical development of white matter and cortical neural function, including abnormal hemispheric lateralization in children with ASD. Analyses also demonstrated a lack of coupling between structure and function in early auditory (M100) as well as later-stage auditory processing (MMF) in ASD. As white-matter microstructure did not explain all the variance in M100 and MMF latency, other aspects of brain structure clearly contribute to age-related changes in M100 and MMF latency. Future multimodal studies examining a broader array of brain structure measures (e.g., gray matter, brainstem white matter, MRS) are needed to more fully understand auditory encoding impairments in children with ASD. Finally, the neural latency measures were found to be of clinical significance, with M100 associated with SRS (but not CELF-4 CLI) indicating that slow auditory processing is an indicator of overall ASD severity, and with MMF latency associated with language performance.

## Author contributions

JB, TR, JE, SL, LB, and EK were involved in study conception and design. All authors were involved in acquisition of MRI, MEG, or clinical scores. JB, TR, JE, LB, EK, JD, and MK were involved in the interpretation or analysis of data. JB, JE, and TR drafted the manuscript. All authors approved the final manuscript.

## Funding

This work was supported by K01MH096091, R01DC008871-07, and the CHOP/UPenn IDDRC grant U54 HD086984.

### Conflict of interest statement

JB is a consultant for McGowan Associates. The other authors declare that the research was conducted in the absence of any commercial or financial relationships that could be construed as a potential conflict of interest. The reviewer AM and the handling Editor declared their shared affiliation, and the handling Editor states that the process nevertheless met the standards of a fair and objective review.
